# Di-μ-chlorido-bis­[(2′-carb­oxybiphen­yl-2-carboxyl­ato-κ*O*)(2,2′:6′,2′′-terpyridine-κ^3^ *N*,*N*′,*N*′′)cadmium(II)] hemihydrate

**DOI:** 10.1107/S1600536809042044

**Published:** 2009-10-23

**Authors:** Wu Zhang, Wen-Juan Li

**Affiliations:** a Institute of Molecular and Crystal Engineering, College of Chemistry and Chemical Engineering, Henan University, Kaifeng 475001, Henan, People’s Republic of China; b Department of Civil and Environmental Engineering, East China Institute of Technology, 56 Xuefu Road, Fuzhou 344000, Jiangxi, People’s Republic of China

## Abstract

In the centrosymmetric dinuclear title compound, [Cd_2_(C_14_H_9_O_4_)_2_Cl_2_(C_15_H_11_N_3_)_2_]·0.5H_2_O, each of the Cd^II^ ions is coordinated by three N atoms from a chelating 2,2′:6′,2′′-terpyridine ligand, two bridging Cl atoms and one O atom of a 2′-carb­oxy-[1,1′-biphen­yl]-2-carboxyl­ate anion. The coordination environment is distorted octa­hedral. In the crystal, inter­molecular O—H⋯O hydrogen bonds link symmetry-related mol­ecules, forming an infinite chain. The half-occupancy water mol­ecule is disordered over two general sites with 0.25 occupancy and is, in turn, disordered over an inversion center.

## Related literature

For background chemistry, see: Meng *et al.* (2004 ▶). For related structures, see: Liu (2009 ▶); Xian *et al.* (2008 ▶); Qu & Li (2008 ▶); Han *et al.* (2008 ▶); Du *et al.* (2008 ▶); Wang *et al.* (2008 ▶); Kurawa *et al.* (2008 ▶); An *et al.* (2009 ▶); Seidel & Oppel (2009 ▶). 
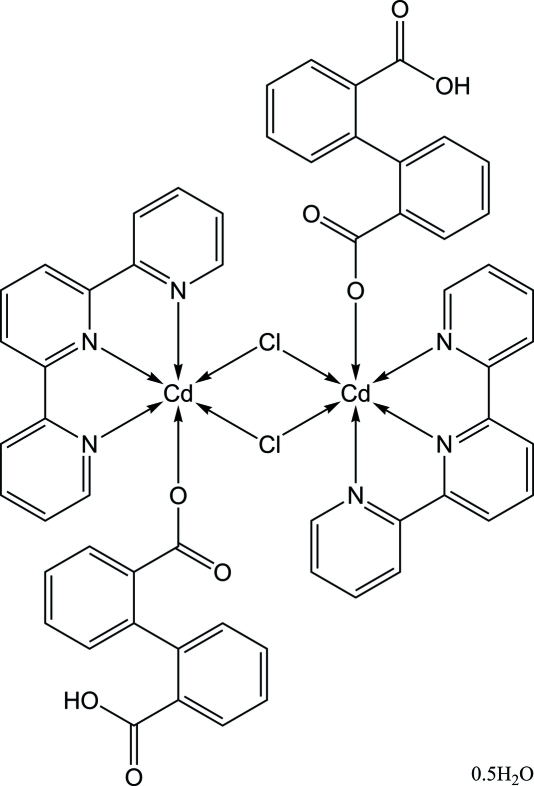


## Experimental

### 

#### Crystal data

[Cd_2_(C_14_H_9_O_4_)_2_Cl_2_(C_15_H_11_N_3_)_2_]·0.5H_2_O
*M* *_r_* = 1253.67
Triclinic, 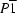

*a* = 9.5673 (18) Å
*b* = 11.575 (2) Å
*c* = 12.565 (2) Å
α = 96.233 (4)°
β = 96.927 (4)°
γ = 108.132 (3)°
*V* = 1297.0 (4) Å^3^
*Z* = 1
Mo *K*α radiation
μ = 0.99 mm^−1^
*T* = 296 K
0.28 × 0.26 × 0.24 mm

#### Data collection

Bruker APEXII CCD area-detector diffractometer
Absorption correction: multi-scan (*SADABS*; Sheldrick, 1996 ▶) *T*_min_ = 0.770, *T*_max_ = 0.798
7084 measured reflections
4529 independent reflections
3969 reflections with *I* > 2σ(*I*)
*R*_int_ = 0.059

#### Refinement

*R*[*F* ^2^ > 2σ(*F* ^2^)] = 0.046
*wR*(*F* ^2^) = 0.124
*S* = 1.01
4529 reflections
353 parameters
H-atom parameters constrained
Δρ_max_ = 1.33 e Å^−3^
Δρ_min_ = −0.98 e Å^−3^

### 

Data collection: *APEX2* (Bruker, 2007 ▶); cell refinement: *SAINT* (Bruker, 2007 ▶); data reduction: *SAINT*; program(s) used to solve structure: *SHELXTL* (Sheldrick, 2008 ▶); program(s) used to refine structure: *SHELXTL*; molecular graphics: *SHELXTL* and *DIAMOND* (Brandenburg & Putz, 1999 ▶); software used to prepare material for publication: *SHELXTL*.

## Supplementary Material

Crystal structure: contains datablocks I, global. DOI: 10.1107/S1600536809042044/nk2006sup1.cif

Structure factors: contains datablocks I. DOI: 10.1107/S1600536809042044/nk2006Isup2.hkl

Additional supplementary materials:  crystallographic information; 3D view; checkCIF report

## Figures and Tables

**Table 1:** Hydrogen-bond geometry (Å, °)

*D*—H⋯*A*	*D*—H	H⋯*A*	*D*⋯*A*	*D*—H⋯*A*
O3—H3*A*⋯O2	0.82	1.68	2.487 (4)	169
O1*W*—H1*WA*⋯O4^i^	0.85	2.41	2.862 (14)	114
O1*W*—H1*WB*⋯O4^ii^	0.85	2.36	3.093 (16)	145

Symmetry codes: (i) 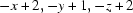; (ii) 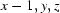.

**Fig. 1.**
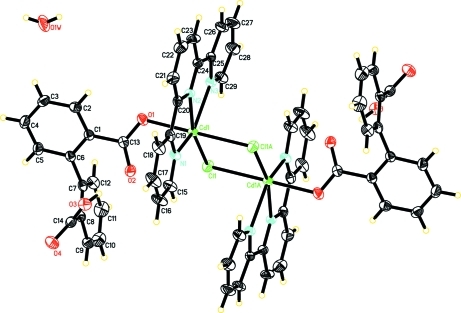
The molecular structure of the title compound with the atom-labelling scheme. Displacement ellipsoids are drawn at the 30% probability level. H atoms are presented as small spheres of arbitrary radius.

**Fig. 2.**
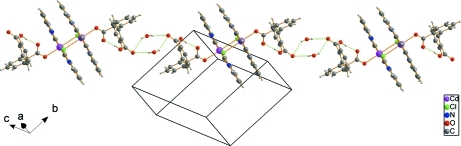
Crystal packing of the title complound. Hydrogen-bond interactions are drawn with dashed lines.

## supplementary crystallographic information

### Comment

Various coordination modes and potential applications in catalysis, fluorescent materials, NLO materials and so on (Meng *et al.* 2004) have been described. Here we report the crystal structure of the title complex prepared from CdCl_2_ and 2'-carboxy-[1,1'-biphenyl]-2-carboxylate ligand (see experimental).

In the centrosymmetric dinuclear title compound, showing in Fig. 1, each of the Cd^II^ ions is coordinated by three N atoms from a chelating 2,2':6',2''-terpyridine ligand, two bridging Cl atoms and one O atom of a 2'-carboxy-[1,1'-biphenyl]-2-carboxylate anion. The coordination environment is distorted octahedral. In the dimeric structure, two Cd^II^ ions are bridged through the Cl atoms, resulting in a planar Cd_2_Cl_2_ core. One of the bridging Cd—Cl bonds is significantly longer than the other. The half-occupancy water molecule is disordered over two general sites with occupancies of 0.25 and 0.25, and is, in turn, disordered over an inversion center.

In the crystal packing, the complex molecules are linked into a one-dimensional infinite chain links supramolecular structure (Fig. 2) by intra- and intermolecular O—H···O, C—H···O and C—H···Cl hydrogen bonding interactions involving the solvent water molecules, the carboxyl group. groups and the chloride (Table 1).

### Experimental

The title complound was synthesized hydrothermally in a Teflon-lined autoclave (25 ml) by heating a mixture of [1,1'-biphenyl]-2,2'-dicarboxylic acid (0.2 mmol), 2,2':6',2''-terpyridine (0.4 mmol) and CdCl_2_.2.5H_2_O (0.2 mmol) in water (10 ml) at 393 K for 3 d. Crystals suitable for X-ray analysis were obtained.

### Refinement

All H atoms were included in calculated positions, with C—H bond lengths fixed at 0.92 Å (carboxyl –COOH), 0.93Å (aryl group) and O—H = 0.85 Å and were refined in the riding-model approximation. *U*_iso_(H) values were calculated at 1.5 *U*_eq_(C) for carboxyl groups and 1.2 *U*_eq_(C) otherwise.

### Crystal data

**Table:**

[Cd_2_(C_14_H_9_O_4_)_2_Cl_2_(C_15_H_11_N_3_)_2_]·0.5H_2_O	*Z* = 1
*M**_r_* = 1253.67	*F*(000) = 629
Triclinic, *P*1	*D*_x_ = 1.605 Mg m^−^^3^
Hall symbol: -P 1	Mo *K*α radiation, λ = 0.71073 Å
*a* = 9.5673 (18) Å	Cell parameters from 4272 reflections
*b* = 11.575 (2) Å	θ = 3.0–27.8°
*c* = 12.565 (2) Å	µ = 0.99 mm^−^^1^
α = 96.233 (4)°	*T* = 296 K
β = 96.927 (4)°	Block, colourless
γ = 108.132 (3)°	0.28 × 0.26 × 0.24 mm
*V* = 1297.0 (4) Å^3^	

### Data collection

**Table:**

Bruker APEXII CCD area-detector diffractometer	4529 independent reflections
Radiation source: sealed tube	3969 reflections with *I* > 2σ(*I*)
graphite	*R*_int_ = 0.059
ω scans	θ_max_ = 25.0°, θ_min_ = 1.7°
Absorption correction: multi-scan (*SADABS*; Sheldrick, 1996)	*h* = −11→6
*T*_min_ = 0.770, *T*_max_ = 0.798	*k* = −11→13
7084 measured reflections	*l* = −13→14

### Refinement

**Table:**

Refinement on *F*^2^	Primary atom site location: structure-invariant direct methods
Least-squares matrix: full	Secondary atom site location: difference Fourier map
*R*[*F*^2^ > 2σ(*F*^2^)] = 0.046	Hydrogen site location: inferred from neighbouring sites
*wR*(*F*^2^) = 0.124	H-atom parameters constrained
*S* = 1.01	*w* = 1/[σ^2^(*F*_o_^2^) + (0.0854*P*)^2^] where *P* = (*F*_o_^2^ + 2*F*_c_^2^)/3
4529 reflections	(Δ/σ)_max_ = 0.001
353 parameters	Δρ_max_ = 1.33 e Å^−^^3^
0 restraints	Δρ_min_ = −0.98 e Å^−^^3^

### Special details

**Table:**

Geometry. All e.s.d.'s (except the e.s.d. in the dihedral angle between two l.s. planes) are estimated using the full covariance matrix. The cell e.s.d.'s are taken into account individually in the estimation of e.s.d.'s in distances, angles and torsion angles; correlations between e.s.d.'s in cell parameters are only used when they are defined by crystal symmetry. An approximate (isotropic) treatment of cell e.s.d.'s is used for estimating e.s.d.'s involving l.s. planes.
Refinement. Refinement of *F*^2^ against ALL reflections. The weighted *R*-factor *wR* and goodness of fit *S* are based on *F*^2^, conventional *R*-factors *R* are based on *F*, with *F* set to zero for negative *F*^2^. The threshold expression of *F*^2^ > σ(*F*^2^) is used only for calculating *R*-factors(gt) *etc*. and is not relevant to the choice of reflections for refinement. *R*-factors based on *F*^2^ are statistically about twice as large as those based on *F*, and *R*- factors based on ALL data will be even larger.

### Fractional atomic coordinates and isotropic or equivalent isotropic displacement parameters (Å^2^)

**Table:**

	*x*	*y*	*z*	*U*_iso_*/*U*_eq_	Occ. (<1)
Cd1	0.89580 (3)	0.91082 (2)	0.60537 (2)	0.03337 (14)	
Cl1	1.04993 (13)	0.86663 (10)	0.46861 (10)	0.0456 (3)	
N1	1.0075 (4)	1.0318 (3)	0.7764 (3)	0.0380 (8)	
N2	0.7167 (4)	0.9594 (3)	0.6971 (3)	0.0315 (7)	
N3	0.6578 (4)	0.8338 (3)	0.4963 (3)	0.0379 (8)	
O1	0.8489 (4)	0.7257 (3)	0.6690 (3)	0.0480 (7)	
O2	1.0910 (4)	0.7855 (3)	0.7351 (2)	0.0473 (7)	
O3	1.2591 (4)	0.7729 (3)	0.8974 (2)	0.0490 (8)	
H3A	1.2060	0.7691	0.8399	0.073*	
O4	1.4450 (4)	0.7087 (4)	0.9518 (3)	0.0715 (11)	
C1	0.9344 (5)	0.5939 (3)	0.7723 (3)	0.0359 (9)	
C2	0.8088 (5)	0.5557 (4)	0.8212 (3)	0.0446 (10)	
H2	0.7380	0.5954	0.8137	0.054*	
C3	0.7870 (6)	0.4608 (4)	0.8802 (4)	0.0500 (12)	
H3	0.7036	0.4381	0.9142	0.060*	
C4	0.8886 (6)	0.3989 (4)	0.8894 (4)	0.0532 (13)	
H4	0.8749	0.3352	0.9304	0.064*	
C5	1.0103 (6)	0.4316 (4)	0.8374 (4)	0.0480 (11)	
H5	1.0761	0.3870	0.8414	0.058*	
C6	1.0381 (5)	0.5304 (3)	0.7786 (3)	0.0362 (9)	
C7	1.1713 (5)	0.5553 (3)	0.7216 (3)	0.0369 (9)	
C8	1.3159 (5)	0.6283 (4)	0.7719 (3)	0.0387 (9)	
C9	1.4367 (5)	0.6326 (4)	0.7207 (4)	0.0503 (11)	
H9	1.5322	0.6786	0.7562	0.060*	
C10	1.4179 (7)	0.5693 (5)	0.6174 (5)	0.0644 (15)	
H10	1.4998	0.5742	0.5828	0.077*	
C11	1.2763 (7)	0.4989 (5)	0.5660 (4)	0.0659 (15)	
H11	1.2627	0.4568	0.4961	0.079*	
C12	1.1544 (6)	0.4905 (4)	0.6176 (4)	0.0484 (11)	
H12	1.0599	0.4410	0.5827	0.058*	
C13	0.9598 (5)	0.7096 (4)	0.7199 (3)	0.0379 (9)	
C14	1.3450 (5)	0.7058 (4)	0.8823 (4)	0.0458 (11)	
C15	1.1542 (6)	1.0720 (4)	0.8099 (4)	0.0524 (12)	
H15	1.2147	1.0521	0.7634	0.063*	
C16	1.2213 (6)	1.1415 (5)	0.9098 (5)	0.0651 (15)	
H16	1.3244	1.1690	0.9299	0.078*	
C17	1.1310 (7)	1.1688 (5)	0.9785 (5)	0.0679 (16)	
H17	1.1723	1.2139	1.0472	0.081*	
C18	0.9788 (6)	1.1290 (4)	0.9454 (4)	0.0549 (12)	
H18	0.9168	1.1472	0.9914	0.066*	
C19	0.9188 (5)	1.0611 (3)	0.8423 (3)	0.0379 (9)	
C20	0.7568 (5)	1.0186 (3)	0.7991 (3)	0.0360 (9)	
C21	0.6505 (6)	1.0399 (4)	0.8586 (4)	0.0490 (11)	
H21	0.6786	1.0824	0.9291	0.059*	
C22	0.5042 (6)	0.9969 (4)	0.8106 (4)	0.0582 (13)	
H22	0.4320	1.0088	0.8499	0.070*	
C23	0.4613 (5)	0.9365 (4)	0.7058 (4)	0.0504 (12)	
H23	0.3617	0.9082	0.6732	0.060*	
C24	0.5730 (4)	0.9188 (3)	0.6493 (4)	0.0367 (9)	
C25	0.5410 (4)	0.8528 (3)	0.5354 (3)	0.0370 (9)	
C26	0.3985 (5)	0.8115 (4)	0.4741 (4)	0.0545 (12)	
H26	0.3191	0.8262	0.5024	0.065*	
C27	0.3772 (6)	0.7487 (5)	0.3713 (5)	0.0657 (15)	
H27	0.2828	0.7202	0.3293	0.079*	
C28	0.4959 (6)	0.7279 (5)	0.3300 (4)	0.0609 (14)	
H28	0.4833	0.6860	0.2602	0.073*	
C29	0.6342 (6)	0.7711 (4)	0.3953 (4)	0.0498 (11)	
H29	0.7144	0.7563	0.3684	0.060*	
O1W	0.4261 (19)	0.4352 (14)	0.9220 (13)	0.082 (5)	0.25
H1WA	0.4041	0.4079	0.9801	0.099*	0.25
H1WB	0.3928	0.4943	0.9141	0.099*	0.25

### Atomic displacement parameters (Å^2^)

**Table:**

	*U*^11^	*U*^22^	*U*^33^	*U*^12^	*U*^13^	*U*^23^
Cd1	0.0282 (2)	0.0394 (2)	0.0330 (2)	0.01333 (13)	0.00468 (13)	0.00142 (13)
Cl1	0.0510 (7)	0.0417 (5)	0.0576 (7)	0.0264 (5)	0.0265 (5)	0.0117 (5)
N1	0.037 (2)	0.0405 (18)	0.0375 (18)	0.0179 (15)	0.0011 (15)	−0.0009 (14)
N2	0.0292 (18)	0.0307 (16)	0.0391 (18)	0.0148 (13)	0.0105 (14)	0.0045 (13)
N3	0.0338 (19)	0.0389 (18)	0.0416 (19)	0.0134 (15)	0.0049 (15)	0.0054 (15)
O1	0.0488 (19)	0.0480 (17)	0.0532 (19)	0.0249 (15)	0.0036 (15)	0.0120 (14)
O2	0.049 (2)	0.0382 (15)	0.0574 (19)	0.0157 (14)	0.0107 (15)	0.0130 (14)
O3	0.052 (2)	0.0532 (18)	0.0356 (16)	0.0137 (16)	0.0057 (14)	−0.0074 (14)
O4	0.056 (2)	0.085 (3)	0.058 (2)	0.010 (2)	−0.0181 (18)	0.0130 (19)
C1	0.039 (2)	0.0345 (19)	0.031 (2)	0.0106 (17)	−0.0013 (17)	0.0006 (16)
C2	0.040 (3)	0.049 (2)	0.042 (2)	0.014 (2)	0.0034 (19)	0.0007 (19)
C3	0.045 (3)	0.051 (3)	0.042 (3)	−0.001 (2)	0.007 (2)	0.006 (2)
C4	0.062 (3)	0.043 (2)	0.048 (3)	0.006 (2)	0.005 (2)	0.016 (2)
C5	0.058 (3)	0.038 (2)	0.047 (3)	0.014 (2)	0.003 (2)	0.0102 (19)
C6	0.039 (2)	0.0317 (19)	0.033 (2)	0.0102 (17)	−0.0032 (17)	0.0005 (16)
C7	0.046 (3)	0.0321 (19)	0.036 (2)	0.0194 (18)	0.0039 (18)	0.0052 (16)
C8	0.041 (2)	0.036 (2)	0.043 (2)	0.0174 (18)	0.0052 (19)	0.0100 (17)
C9	0.041 (3)	0.047 (2)	0.070 (3)	0.022 (2)	0.012 (2)	0.015 (2)
C10	0.074 (4)	0.057 (3)	0.079 (4)	0.034 (3)	0.037 (3)	0.012 (3)
C11	0.091 (5)	0.058 (3)	0.058 (3)	0.036 (3)	0.029 (3)	−0.007 (2)
C12	0.057 (3)	0.043 (2)	0.044 (2)	0.021 (2)	0.002 (2)	−0.0056 (19)
C13	0.046 (3)	0.040 (2)	0.032 (2)	0.020 (2)	0.0075 (18)	0.0031 (17)
C14	0.043 (3)	0.048 (2)	0.037 (2)	0.003 (2)	0.001 (2)	0.0098 (19)
C15	0.044 (3)	0.055 (3)	0.055 (3)	0.021 (2)	−0.005 (2)	−0.007 (2)
C16	0.047 (3)	0.057 (3)	0.078 (4)	0.019 (2)	−0.024 (3)	−0.020 (3)
C17	0.075 (4)	0.059 (3)	0.061 (3)	0.035 (3)	−0.026 (3)	−0.023 (3)
C18	0.068 (3)	0.055 (3)	0.044 (3)	0.033 (3)	−0.002 (2)	−0.008 (2)
C19	0.049 (3)	0.0319 (19)	0.036 (2)	0.0203 (18)	0.0048 (19)	0.0027 (16)
C20	0.043 (2)	0.0335 (19)	0.036 (2)	0.0175 (17)	0.0098 (18)	0.0083 (16)
C21	0.057 (3)	0.052 (3)	0.047 (3)	0.027 (2)	0.022 (2)	0.005 (2)
C22	0.058 (3)	0.058 (3)	0.070 (3)	0.027 (2)	0.035 (3)	0.009 (3)
C23	0.030 (2)	0.056 (3)	0.070 (3)	0.019 (2)	0.018 (2)	0.007 (2)
C24	0.032 (2)	0.0327 (19)	0.050 (2)	0.0146 (16)	0.0097 (18)	0.0098 (17)
C25	0.031 (2)	0.0304 (19)	0.050 (2)	0.0097 (16)	0.0021 (18)	0.0116 (17)
C26	0.034 (3)	0.058 (3)	0.072 (3)	0.020 (2)	−0.003 (2)	0.007 (2)
C27	0.042 (3)	0.071 (3)	0.067 (4)	0.009 (2)	−0.018 (3)	−0.001 (3)
C28	0.056 (3)	0.058 (3)	0.053 (3)	0.011 (2)	−0.012 (2)	−0.010 (2)
C29	0.048 (3)	0.051 (3)	0.048 (3)	0.019 (2)	−0.002 (2)	−0.004 (2)
O1W	0.094 (13)	0.085 (11)	0.094 (12)	0.060 (10)	0.012 (10)	0.039 (10)

### Geometric parameters (Å, °)

**Table:**

Cd1—O1	2.301 (3)	C9—C10	1.381 (7)
Cd1—N2	2.349 (3)	C9—H9	0.9300
Cd1—N3	2.362 (3)	C10—C11	1.380 (8)
Cd1—N1	2.363 (3)	C10—H10	0.9300
Cd1—Cl1	2.5058 (11)	C11—C12	1.384 (7)
Cd1—Cl1^i^	2.7520 (12)	C11—H11	0.9300
Cl1—Cd1^i^	2.7520 (11)	C12—H12	0.9300
N1—C15	1.331 (6)	C15—C16	1.378 (7)
N1—C19	1.345 (5)	C15—H15	0.9300
N2—C20	1.336 (5)	C16—C17	1.372 (8)
N2—C24	1.345 (5)	C16—H16	0.9300
N3—C25	1.342 (5)	C17—C18	1.379 (8)
N3—C29	1.347 (6)	C17—H17	0.9300
O1—C13	1.247 (5)	C18—C19	1.394 (6)
O2—C13	1.266 (5)	C18—H18	0.9300
O3—C14	1.312 (6)	C19—C20	1.484 (6)
O3—H3A	0.8200	C20—C21	1.398 (6)
O4—C14	1.204 (6)	C21—C22	1.367 (7)
C1—C2	1.387 (6)	C21—H21	0.9300
C1—C6	1.405 (6)	C22—C23	1.372 (7)
C1—C13	1.521 (6)	C22—H22	0.9300
C2—C3	1.369 (6)	C23—C24	1.405 (6)
C2—H2	0.9300	C23—H23	0.9300
C3—C4	1.377 (7)	C24—C25	1.496 (6)
C3—H3	0.9300	C25—C26	1.394 (6)
C4—C5	1.374 (7)	C26—C27	1.371 (7)
C4—H4	0.9300	C26—H26	0.9300
C5—C6	1.403 (6)	C27—C28	1.379 (8)
C5—H5	0.9300	C27—H27	0.9300
C6—C7	1.502 (6)	C28—C29	1.383 (7)
C7—C12	1.399 (6)	C28—H28	0.9300
C7—C8	1.405 (6)	C29—H29	0.9300
C8—C9	1.380 (6)	O1W—H1WA	0.8500
C8—C14	1.512 (6)	O1W—H1WB	0.8500
			
O1—Cd1—N2	91.38 (11)	C10—C11—H11	119.8
O1—Cd1—N3	88.97 (11)	C12—C11—H11	119.8
N2—Cd1—N3	69.18 (12)	C11—C12—C7	120.9 (5)
O1—Cd1—N1	94.88 (12)	C11—C12—H12	119.5
N2—Cd1—N1	69.08 (12)	C7—C12—H12	119.5
N3—Cd1—N1	138.15 (12)	O1—C13—O2	124.4 (4)
O1—Cd1—Cl1	96.17 (8)	O1—C13—C1	117.7 (4)
N2—Cd1—Cl1	166.28 (9)	O2—C13—C1	117.7 (4)
N3—Cd1—Cl1	99.44 (9)	O4—C14—O3	121.6 (5)
N1—Cd1—Cl1	121.40 (9)	O4—C14—C8	121.8 (5)
O1—Cd1—Cl1^i^	179.22 (8)	O3—C14—C8	116.6 (4)
N2—Cd1—Cl1^i^	87.87 (8)	N1—C15—C16	123.5 (5)
N3—Cd1—Cl1^i^	90.99 (8)	N1—C15—H15	118.2
N1—Cd1—Cl1^i^	84.62 (9)	C16—C15—H15	118.2
Cl1—Cd1—Cl1^i^	84.61 (3)	C17—C16—C15	117.8 (5)
Cd1—Cl1—Cd1^i^	95.39 (3)	C17—C16—H16	121.1
C15—N1—C19	118.9 (4)	C15—C16—H16	121.1
C15—N1—Cd1	122.7 (3)	C16—C17—C18	119.7 (5)
C19—N1—Cd1	118.4 (3)	C16—C17—H17	120.1
C20—N2—C24	120.8 (3)	C18—C17—H17	120.1
C20—N2—Cd1	119.6 (3)	C17—C18—C19	119.3 (5)
C24—N2—Cd1	119.4 (3)	C17—C18—H18	120.3
C25—N3—C29	118.3 (4)	C19—C18—H18	120.3
C25—N3—Cd1	119.3 (3)	N1—C19—C18	120.7 (4)
C29—N3—Cd1	122.4 (3)	N1—C19—C20	116.6 (3)
C13—O1—Cd1	115.1 (3)	C18—C19—C20	122.7 (4)
C14—O3—H3A	109.5	N2—C20—C21	120.8 (4)
C2—C1—C6	119.7 (4)	N2—C20—C19	115.9 (3)
C2—C1—C13	117.9 (4)	C21—C20—C19	123.3 (4)
C6—C1—C13	122.2 (4)	C22—C21—C20	118.4 (4)
C3—C2—C1	121.2 (4)	C22—C21—H21	120.8
C3—C2—H2	119.4	C20—C21—H21	120.8
C1—C2—H2	119.4	C21—C22—C23	121.5 (4)
C2—C3—C4	120.1 (5)	C21—C22—H22	119.3
C2—C3—H3	120.0	C23—C22—H22	119.3
C4—C3—H3	120.0	C22—C23—C24	117.7 (4)
C5—C4—C3	119.6 (4)	C22—C23—H23	121.2
C5—C4—H4	120.2	C24—C23—H23	121.2
C3—C4—H4	120.2	N2—C24—C23	120.8 (4)
C4—C5—C6	121.8 (4)	N2—C24—C25	116.1 (3)
C4—C5—H5	119.1	C23—C24—C25	123.1 (4)
C6—C5—H5	119.1	N3—C25—C26	121.9 (4)
C5—C6—C1	117.5 (4)	N3—C25—C24	115.8 (4)
C5—C6—C7	117.3 (4)	C26—C25—C24	122.3 (4)
C1—C6—C7	125.1 (4)	C27—C26—C25	118.9 (5)
C12—C7—C8	117.8 (4)	C27—C26—H26	120.6
C12—C7—C6	118.3 (4)	C25—C26—H26	120.6
C8—C7—C6	123.5 (4)	C26—C27—C28	120.0 (5)
C9—C8—C7	120.5 (4)	C26—C27—H27	120.0
C9—C8—C14	117.7 (4)	C28—C27—H27	120.0
C7—C8—C14	121.8 (4)	C27—C28—C29	118.1 (5)
C8—C9—C10	120.9 (5)	C27—C28—H28	120.9
C8—C9—H9	119.5	C29—C28—H28	120.9
C10—C9—H9	119.5	N3—C29—C28	122.9 (5)
C11—C10—C9	119.3 (5)	N3—C29—H29	118.6
C11—C10—H10	120.4	C28—C29—H29	118.6
C9—C10—H10	120.4	H1WA—O1W—H1WB	109.5
C10—C11—C12	120.5 (5)		
			
O1—Cd1—Cl1—Cd1^i^	−179.90 (8)	C8—C9—C10—C11	−1.5 (8)
N2—Cd1—Cl1—Cd1^i^	57.1 (3)	C9—C10—C11—C12	−0.7 (8)
N3—Cd1—Cl1—Cd1^i^	90.11 (9)	C10—C11—C12—C7	1.6 (8)
N1—Cd1—Cl1—Cd1^i^	−80.34 (10)	C8—C7—C12—C11	−0.4 (7)
Cl1^i^—Cd1—Cl1—Cd1^i^	0.0	C6—C7—C12—C11	−172.7 (4)
O1—Cd1—N1—C15	94.5 (4)	Cd1—O1—C13—O2	−1.7 (5)
N2—Cd1—N1—C15	−175.9 (4)	Cd1—O1—C13—C1	175.0 (3)
N3—Cd1—N1—C15	−171.6 (3)	C2—C1—C13—O1	−39.5 (5)
Cl1—Cd1—N1—C15	−5.8 (4)	C6—C1—C13—O1	144.7 (4)
Cl1^i^—Cd1—N1—C15	−86.1 (3)	C2—C1—C13—O2	137.4 (4)
O1—Cd1—N1—C19	−85.5 (3)	C6—C1—C13—O2	−38.4 (5)
N2—Cd1—N1—C19	4.1 (3)	C9—C8—C14—O4	−45.6 (6)
N3—Cd1—N1—C19	8.5 (4)	C7—C8—C14—O4	135.2 (5)
Cl1—Cd1—N1—C19	174.3 (2)	C9—C8—C14—O3	132.2 (4)
Cl1^i^—Cd1—N1—C19	93.9 (3)	C7—C8—C14—O3	−47.1 (6)
O1—Cd1—N2—C20	89.0 (3)	C19—N1—C15—C16	0.7 (7)
N3—Cd1—N2—C20	177.4 (3)	Cd1—N1—C15—C16	−179.2 (4)
N1—Cd1—N2—C20	−5.7 (3)	N1—C15—C16—C17	1.0 (8)
Cl1—Cd1—N2—C20	−147.5 (3)	C15—C16—C17—C18	−1.4 (8)
Cl1^i^—Cd1—N2—C20	−90.7 (3)	C16—C17—C18—C19	0.2 (8)
O1—Cd1—N2—C24	−85.8 (3)	C15—N1—C19—C18	−2.1 (6)
N3—Cd1—N2—C24	2.6 (3)	Cd1—N1—C19—C18	177.9 (3)
N1—Cd1—N2—C24	179.5 (3)	C15—N1—C19—C20	177.5 (4)
Cl1—Cd1—N2—C24	37.7 (5)	Cd1—N1—C19—C20	−2.6 (4)
Cl1^i^—Cd1—N2—C24	94.5 (3)	C17—C18—C19—N1	1.6 (7)
O1—Cd1—N3—C25	91.9 (3)	C17—C18—C19—C20	−177.9 (4)
N2—Cd1—N3—C25	0.1 (3)	C24—N2—C20—C21	−0.1 (6)
N1—Cd1—N3—C25	−4.2 (4)	Cd1—N2—C20—C21	−174.8 (3)
Cl1—Cd1—N3—C25	−172.0 (3)	C24—N2—C20—C19	−178.8 (3)
Cl1^i^—Cd1—N3—C25	−87.3 (3)	Cd1—N2—C20—C19	6.4 (4)
O1—Cd1—N3—C29	−88.3 (3)	N1—C19—C20—N2	−2.5 (5)
N2—Cd1—N3—C29	179.9 (4)	C18—C19—C20—N2	177.0 (4)
N1—Cd1—N3—C29	175.6 (3)	N1—C19—C20—C21	178.8 (4)
Cl1—Cd1—N3—C29	7.8 (3)	C18—C19—C20—C21	−1.7 (6)
Cl1^i^—Cd1—N3—C29	92.5 (3)	N2—C20—C21—C22	1.2 (6)
N2—Cd1—O1—C13	−123.7 (3)	C19—C20—C21—C22	179.8 (4)
N3—Cd1—O1—C13	167.1 (3)	C20—C21—C22—C23	−1.5 (7)
N1—Cd1—O1—C13	−54.6 (3)	C21—C22—C23—C24	0.8 (7)
Cl1—Cd1—O1—C13	67.8 (3)	C20—N2—C24—C23	−0.7 (6)
C6—C1—C2—C3	3.2 (6)	Cd1—N2—C24—C23	174.1 (3)
C13—C1—C2—C3	−172.7 (4)	C20—N2—C24—C25	−179.4 (3)
C1—C2—C3—C4	−1.9 (7)	Cd1—N2—C24—C25	−4.7 (4)
C2—C3—C4—C5	−1.1 (7)	C22—C23—C24—N2	0.4 (6)
C3—C4—C5—C6	2.8 (7)	C22—C23—C24—C25	179.0 (4)
C4—C5—C6—C1	−1.4 (6)	C29—N3—C25—C26	−1.0 (6)
C4—C5—C6—C7	−177.8 (4)	Cd1—N3—C25—C26	178.8 (3)
C2—C1—C6—C5	−1.6 (6)	C29—N3—C25—C24	177.8 (4)
C13—C1—C6—C5	174.2 (4)	Cd1—N3—C25—C24	−2.3 (4)
C2—C1—C6—C7	174.5 (4)	N2—C24—C25—N3	4.6 (5)
C13—C1—C6—C7	−9.7 (6)	C23—C24—C25—N3	−174.2 (4)
C5—C6—C7—C12	85.8 (5)	N2—C24—C25—C26	−176.6 (4)
C1—C6—C7—C12	−90.3 (5)	C23—C24—C25—C26	4.7 (6)
C5—C6—C7—C8	−86.0 (5)	N3—C25—C26—C27	0.5 (7)
C1—C6—C7—C8	97.9 (5)	C24—C25—C26—C27	−178.2 (4)
C12—C7—C8—C9	−1.7 (6)	C25—C26—C27—C28	−0.2 (8)
C6—C7—C8—C9	170.2 (4)	C26—C27—C28—C29	0.5 (8)
C12—C7—C8—C14	177.5 (4)	C25—N3—C29—C28	1.2 (7)
C6—C7—C8—C14	−10.6 (6)	Cd1—N3—C29—C28	−178.6 (4)
C7—C8—C9—C10	2.7 (7)	C27—C28—C29—N3	−1.0 (8)
C14—C8—C9—C10	−176.6 (4)		

Symmetry codes: (i) −*x*+2, −*y*+2, −*z*+1.

### Hydrogen-bond geometry (Å, °)

**Table:**

*D*—H···*A*	*D*—H	H···*A*	*D*···*A*	*D*—H···*A*
O3—H3A···O2	0.82	1.68	2.487 (4)	169
O1W—H1WA···O4^ii^	0.85	2.41	2.862 (14)	114
O1W—H1WB···O4^iii^	0.85	2.36	3.093 (16)	145
C16—H16···O4^iv^	0.93	2.43	3.284 (6)	153
C21—H21···O3^v^	0.93	2.49	3.405 (6)	169
C26—H26···Cl1^iii^	0.93	2.75	3.579 (5)	149

Symmetry codes: (ii) −*x*+2, −*y*+1, −*z*+2; (iii) *x*−1, *y*, *z*; (iv) −*x*+3, −*y*+2, −*z*+2; (v) −*x*+2, −*y*+2, −*z*+2.
